# The epiphany derived from T-cell–inflamed profiles: Pan-cancer characterization of CD8A as a biomarker spanning clinical relevance, cancer prognosis, immunosuppressive environment, and treatment responses

**DOI:** 10.3389/fgene.2022.974416

**Published:** 2022-08-11

**Authors:** Decao Niu, Yifeng Chen, Hua Mi, Zengnan Mo, Guijian Pang

**Affiliations:** ^1^ Department of Urology, The First Affiliated Hospital of Guangxi Medical University, Nanning, China; ^2^ Center for Genomic and Personalized Medicine, Guangxi Key Laboratory for Genomic and Personalized Medicine, Guangxi Collaborative Innovation Center for Genomic and Personalized Medicine, Guangxi Medical University, Nanning, China; ^3^ Department of Urology, Guangdong Second Provincial General Hospital, Guangzhou, China; ^4^ Department of Urology, The First People’s Hospital of Yulin, Yulin, China

**Keywords:** pan-cancer, CD8A, prognosis, biomarker, tumor microenvironment, immunotherapy

## Abstract

CD8A encodes the CD8 alpha chain of αβT cells, which has been proposed as a quantifiable indicator for the assessment of CD8^+^ cytotoxic T lymphocytes (CTLs) recruitment or activity and a robust biomarker for anti-PD-1/PD-L1 therapy responses. Nonetheless, the lack of research into the role of CD8A in tumor microenvironment predisposes to limitations in its clinical utilization. In the presented study, multiple computational tools were used to investigate the roles of CD8A in the pan-cancer study, revealing its essential associations with tumor immune infiltration, immunosuppressive environment formation, cancer progression, and therapy responses. Based on the pan-cancer cohorts of the Cancer Genome Atlas (TCGA) database, our results demonstrated the distinctive CD8A expression patterns in cancer tissues and its close associations with the prognosis and disease stage of cancer. We then found that CD8A was correlated with six major immune cell types, and immunosuppressive cells in multiple cancer types. Besides, epigenetic modifications of CD8A were related to CTL levels and T cell dysfunctional states, thereby affecting survival outcomes of specific cancer types. After that, we explored the co-occurrence patterns of CD8A mutation, thus identifying RMND5A, RNF103-CHMP3, CHMP3, CD8B, MRPL35, MAT2A, RGPD1, RGPD2, REEP1, and ANAPC1P1 genes, which co-occurred mutations with CD8A, and are concomitantly implicated in the regulation of cancer-related pathways. Finally, we tested CD8A as a therapeutic biomarker for multiple antitumor agents’ or compounds’ responsiveness on various cancer cell lines and cancer cohorts. Our findings denoted the underlying mechanics of CD8A in reflecting the T-cell-inflamed profiles, which has potential as a biomarker in cancer diagnosis, prognosis, and therapeutic responses.

## Introduction

As knowledge of underlying mechanisms of cancer revolutionarily progressed, it has now been considered a genetic disease (Chen and Mellman, 2017), characterized by genetic mutations that activate oncogenic drivers, and epigenetic regulations independent of genome reprogramming (Hanahan, 2022). The illustrative perspectives provided by multi-omic technologies are intended to illuminate the myriad manifestations of cancer during malignant development and metastasis, thus applying to cancer precision treatment, particularly immunotherapy. The advent of immune checkpoint blockade (ICB) has revolutionized therapeutic profiles of cancer, which reinvigorate antitumor responses by blocking co-inhibitory signaling pathways, thus contributing to the elimination of cancer cells via the release of cytokines, and cytotoxic granules of effector T cells (Sharma and Allison, 2015; Darvin et al., 2018). Since first ICB targeting cytotoxic T lymphocyte antigen 4 (CTLA-4), ipilimumab, garnered approval for the treatment of melanoma in 2011 (Hodi et al., 2010; Robert et al., 2011), ICBs targeting programmed cell death protein 1/programmed cell death ligand 1 (PD-1/PD-L1) are also being investigated in the clinical trials, manifesting compelling clinical effectiveness (Ansell et al., 2015; Borghaei et al., 2015; Larkin et al., 2015). Partial patients do not benefit from ICB, exhibiting *de novo* or adaptive resistance (Gnjatic et al., 2017). In contrast, the molecular underpinnings of such resistance remain unknown, providing a rationale for identifying predictive biomarkers and tailoring immunotherapeutic regimens appropriately. Likewise, other canonical biomarkers, such as tumor mutational burden (TMB) (Snyder et al., 2014), microsatellite instability (MSI) (Snyder et al., 2014), tumor-infiltrating lymphocytes (TILs) (Cogdill et al., 2017), and epigenetic signatures (Marwitz et al., 2017), have been intensively studied individually or in combination to identify more specific predictors of ICB efficacy, indicating unmet research and clinical need. Notably, most biomarkers analyses are limited to particular tumor types, making them lack generalizability to other clinical populations. Besides, underlying mechanisms and predictive accuracy of the aforementioned biomarkers are only partially understood; robust predictive options are further limited.

The dynamic alterations of tumor microenvironment (TME) components can influence cancer progression, and therapeutic outcomes, which has been well appreciated and documented. (Binnewies et al., 2018; Chen et al., 2021). Thus, therapeutically targeting the TME as an intervention for mounting active immune responses or relieving immunosuppressive environments has been proposed (Bejarano et al., 2021). Specifically, TME could be divided into three major immune profiles, including immune desert, immune excluded, and immune inflamed (Chen and Mellman, 2017), based upon the infiltration levels and types of immune cell. Indeed, the clinical response rate to ICBs is generally higher in the immune-inflamed profile (Herbst et al., 2014; Hegde et al., 2016), including pre-existing immune cell (Darvin et al., 2018), detectable pro-inflammatory and effector cytokines (Fehrenbacher et al., 2016; Rosenberg et al., 2016). T-cell-inflamed phenotype, characterized by elevated type I interferon-related transcriptional profiling accompanied by promigratory chemokines contributing to recruitment of activated CD8^+^ effector T cells (Trujillo et al., 2018), has become a significant clinical and research interest owing to its close association with better cancer patient survival outcomes (Bruni et al., 2020), and enhanced responses to ICBs (Chen and Mellman, 2017; Gruber et al., 2020).

To date, the cellular actors of CD8^+^ effector T cells or cytotoxic T lymphocytes (CTLs) implicated in the tumor immunity cycle are well described, with evidence revealing its paradoxical effects in TME. In addition to acting as a preferred immune cell type targeting tumor cells and serving as a frontline defense against tumor progression (Farhood et al., 2019; Raskov et al., 2021), infiltration of CTLs and high levels of IFN-γ secretion in the microenvironment synergistically attribute to the upregulation of transcripts encoding indoleamine-2,3-dioxygenase (IDO), PD-L1, and forkhead box protein 3 (FOXP3), thus yielding the establishments of the immunosuppressive microenvironment, modification of TME metabolism, and recruitment of FOXP3^+^ regulatory T cells (Tregs) (Spranger et al., 2013; Trujillo et al., 2018; Olson and Luke, 2019). Thus, these illustrative snapshots raise the intriguing possibility that T cell-inflamed genes or signatures can serve as a paradigm-shifting breakpoint for delineating mechanisms of the immunosuppressive microenvironment, and tailoring therapeutic regimens to overcome immunotherapy resistance.

**FIGURE 1:**
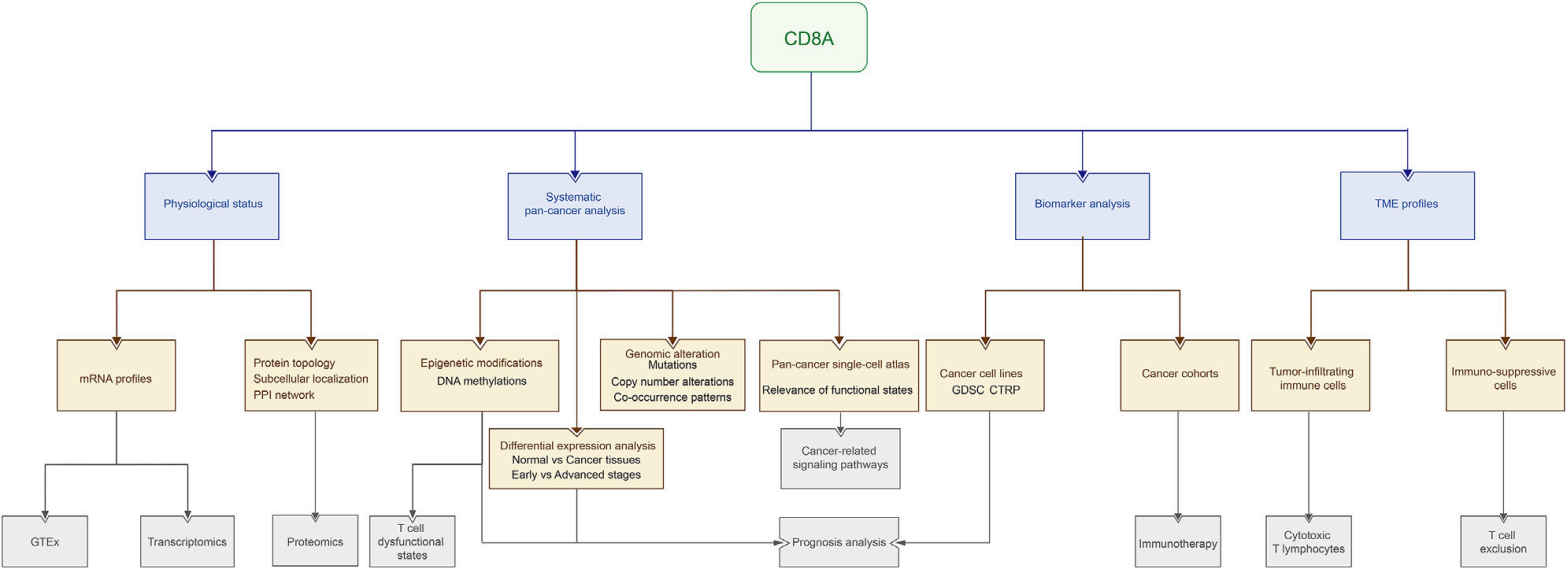
The workflow of the presented work.

CD8A encodes the CD8 alpha chain of the αβT cells, proposed as a quantifiable indicator for CD8^+^ CTL recruitment or activity assessments and a robust biomarker for responses to anti-PD-1/PD-L1 therapy (Ock et al., 2016). Despite being expressed in various immune cell types, CTLs present a predominant expression level of CD8A (Ma K. et al., 2020), which could be a direct indication of pre-existing antitumor immunity with tumor-infiltrating CTLs in TME (Lei et al., 2021). As previously described, the varying function of CTLs makes CD8A a promising gene in predicting cancer patient survival outcomes and a potential biomarker in assessing antitumor agent responses. Nonetheless, the lack of investigation into the role of CD8A in TME predisposed to limitations in its clinical use, necessitating further research into its underlying mechanisms in the pan-cancer cohort. In this work, our purpose here is to delineate the roles of CD8A in pan-cancer cohort, whereby revealing its essential associations with tumor immune infiltration, immunosuppressive environment formation, cancer progression, and therapy responses using multiple computational tools. Besides, the results mentioned above were validated via cancer cell lines and cancer cohorts, together manifesting the underlying mechanics of CD8A in reflecting the T-cell-inflamed profiles, and its potential as a biomarker in cancer diagnosis, prognosis, and therapeutic responses. We then investigated CD8A epigenetic modifications and their relationship to T cell dysfunctional states. Next, we dissected genomic alteration profiles along with co-occurrence patterns of CD8A mutations, and its relevance across functional states in single-cell resolution. The workflow of presented work is depicted in Figure 1.

## Materials and methods

### Raw data acquisition

Transcriptomic information and corresponding clinical data for 33 types of pan-cancer cohorts were downloaded from The Cancer Genome Atlas (TCGA) database via the University of California Santa Cruz Xena (UCSC Xena; https://xena.ucsc.edu/).

### Characterization of online analytical tools

GeneCards (www.genecards.org) is a comprehensive compendium of information on human genes, also providing visualization of transcriptomic expression profiles in healthy and cancer tissues (Safran et al., 2010). Protter (http://wlab.ethz.ch/protter) is an online tool supporting proteomic data analysis and hypothesis generation via visualization for annotated sequence features in the context of protein topology (Omasits et al., 2014). GPS-Prot (http://gpsprot.org/index.php) is a web-based platform for integrating and constructing the protein-protein interaction (PPI) network (Fahey et al., 2011). Human Protein Atlas (HPA) (https://www.proteinatlas.org) is an interactive proteome database including multi-omics information at the tissue and organ levels of the human body, allowing for spatial protein localization down to the single-cell level (Uhlén et al., 2015). OPENTARGETS (https://www.targetvalidation.org/) is an integration platform providing evidence about the correlations of potential drug targets with human diseases (Koscielny et al., 2017). Tumor IMmune Estimation Resource (TIMER2.0) (http://timer.cistrome.org/) is a web server supporting robust estimation of immune infiltration levels based on TCGA using multiple algorithms (Li et al., 2020). Tumor Immune Dysfunction and Exclusion (TIDE) (http://tide.dfci.harvard.edu/) is a web application integrating the expression profiles of T cell dysfunction and exclusion, thereby modeling immune evasion of tumor cells, and has the potential to predict response of ICBs (Jiang et al., 2018). The University of Alabama Cancer database (UALCAN) (http://ualcan.path.uab.edu/) is a user-friendly web portal that performs analysis of gene expression data of various cancer types in TCGA (Chandrashekar et al., 2017). cBioPortal (http://www.cbioportal.org/) is an online tool for researchers to analyze multidimensional cancer genomics data interactively (Gao et al., 2013). Gene Set Cancer Analysis (GSCALite) (http://bioinfo.life.hust.edu.cn/web/GSCALite/) is an accessible webserver to dynamically analyze and visualize gene sets in cancer as well as sensitivity correlation for the drug (Liu C. J. et al., 2018). The ROC plotter (http://www.rocplot.org/) is an online tool designed to identify novel biomarkers that can predict therapy responses for patients treated with chemotherapy, hormonal therapy, and targeted therapy by analyzing the selected genes in multiple independent datasets (Fekete and Győrffy, 2019). Cancer single-cell state atlas (CancerSEA) (http://biocc.hrbmu.edu.cn/CancerSEA/) is a dedicated database that investigates distinct functional states of cancer cells at the single-cell resolutions (Yuan et al., 2019).

### Physiological and expression patterns of CD8A

To analyze transcriptomic expression patterns of CD8A under physiological conditions, we used GeneCards database to visualize mRNA levels of CD8A in different organs of the human body. Protter database was used to present the topology structure of CD8A, and intracellular localization of CD8A in varying cell lines was displayed using HPA database, followed by the application of OPENTAGET, thereby identifying disease phenotypes associated with CD8A using 0.1 as the minimum score. Thereafter, differential expression analysis of CD8A between tumor and normal control was analyzed based on TCGA using the “ggpubr” (Kassambara, 2020) package in R language.

### Correlation analysis of CD8A with prognosis and clinical stages

Kaplan-Meier analysis was conducted to analyze associations between CD8A and overall survival of pan-cancer patients in TCGA database using “survival” (Therneau, 2015) and “survminer” (Kassambara et al., 2018) R packages. Then, using the Wilcox test, the differential analysis of CD8A expression levels between early and late clinical stages were analyzed and visualized through R packages “limma” (Ritchie et al., 2015) and “ggpubr” (Kassambara, 2020).

### Correlation analysis of CD8A expression with infiltrating immune cells, and immunosuppressive cell profiles in TME

We used TIMER2 database to delineate associations between CD8A and canonical infiltrating immune cell types in TME. Besides, we investigated associations between CD8A and immunosuppressive cells, accounting for T cell exclusion. Spearman’s correlation analyses were conducted in the processes above and visualized via “RColorBrewer” (Erich, 2014) R packages.

### Epigenetic methylation analysis of CD8A

UALCAN database was used to conduct a differential analysis of methylation levels between tumor and normal tissues. In reference to annotations in UALCAN database (Shinawi et al., 2013; Men et al., 2017), we defined hypermethylation as a beta value of 0.7–0.5, while a beta value of 0.25–0.3 was considered hypomethylation. Besides, the Cox proportional hazard (Cox-PH) model regression based upon the TIDE database was used to reflect the combined effects of CD8A methylation and CTL level on the prognosis of cancer patients, thereby revealing the associations between epigenetic methylation and dysfunctional T cell phenotypes.

### Genomic alteration profiles and co-occurrence patterns of CD8A

We used cBioPortal database to visualize CD8A alteration profiles of TCGA pan-cancer atlas studies, thus identifying co-occurrence patterns with CD8A mutation, with a log ratio of >6, *p-*value and Q-value less than 10^−10^, then presenting the frequency and mutation type of these co-occurred genes with CD8A using waterfall plots and histograms, respectively. Furthermore, GSCALite was used to assess the role of above-mentioned genes in regulating cancer-related signaling pathways across different cancer types.

### Single-cell analysis for providing CD8A repertoires in TME

We used CancerSEA database to investigate the role of CD8A in TME at single-cell resolution and its correlation with malignant phenotype and functional states, thereby providing CD8A repertoires in TME.

### Biomarker analysis of CD8A as an indicator of response to antitumor compounds and agents

We used GSCALite database, which contains drug response data toward human cancer cell lines from the Genomics of Drug Sensitivity in Cancer (GDSC) and Cancer Therapeutic Response Portal (CTRP), to examine relationships between CD8A and the sensitivity of various antitumor agents or compounds to tumor cell lines. 50% inhibitory concentration (IC_50_) was used as the index reflecting the sensitivity of the small molecules and compounds. Additionally, we evaluated the predictive ability of CD8A for immunotherapy efficacy compared with other canonical biomarker signatures using the multiple cancer cohorts treated with ICB in TIDE database and further analyzed the associations between CD8A expression and CTL levels. Furthermore, ROC plotter database was used to assess the feasibility of CD8A in assessing therapeutic responses to various antitumor drugs in clinical cancer cohorts such as glioblastoma multiforme (GBM) cohorts treated with chemotherapy, invasive breast carcinoma (BRCA) cohorts treated with anti-HER2 antibody and chemotherapeutics, and ovarian cancer cohorts treated with targeted drugs and chemotherapeutics. The receiver operating characteristic (ROC) curves was used to present predictive ability of CD8A in predicting efficacy of various antitumor agents in the above analysis.

### Statistical analysis

Mann-Whitney test was used to compare transcriptomic patterns of CD8A between cancer and normal control. Furthermore, Kaplan-Meier analysis determined the relationship between CD8A and pan-cancer patient prognosis. *p* < 0.05 was indicative of statistically significant.

### Ethic statement

Our study was based on the online database, and ethics approval was not required.

## Results

### Protein topology, subcellular localization, PPI network analysis, and transcriptome expression of CD8A under the physiological status

**FIGURE 2:**
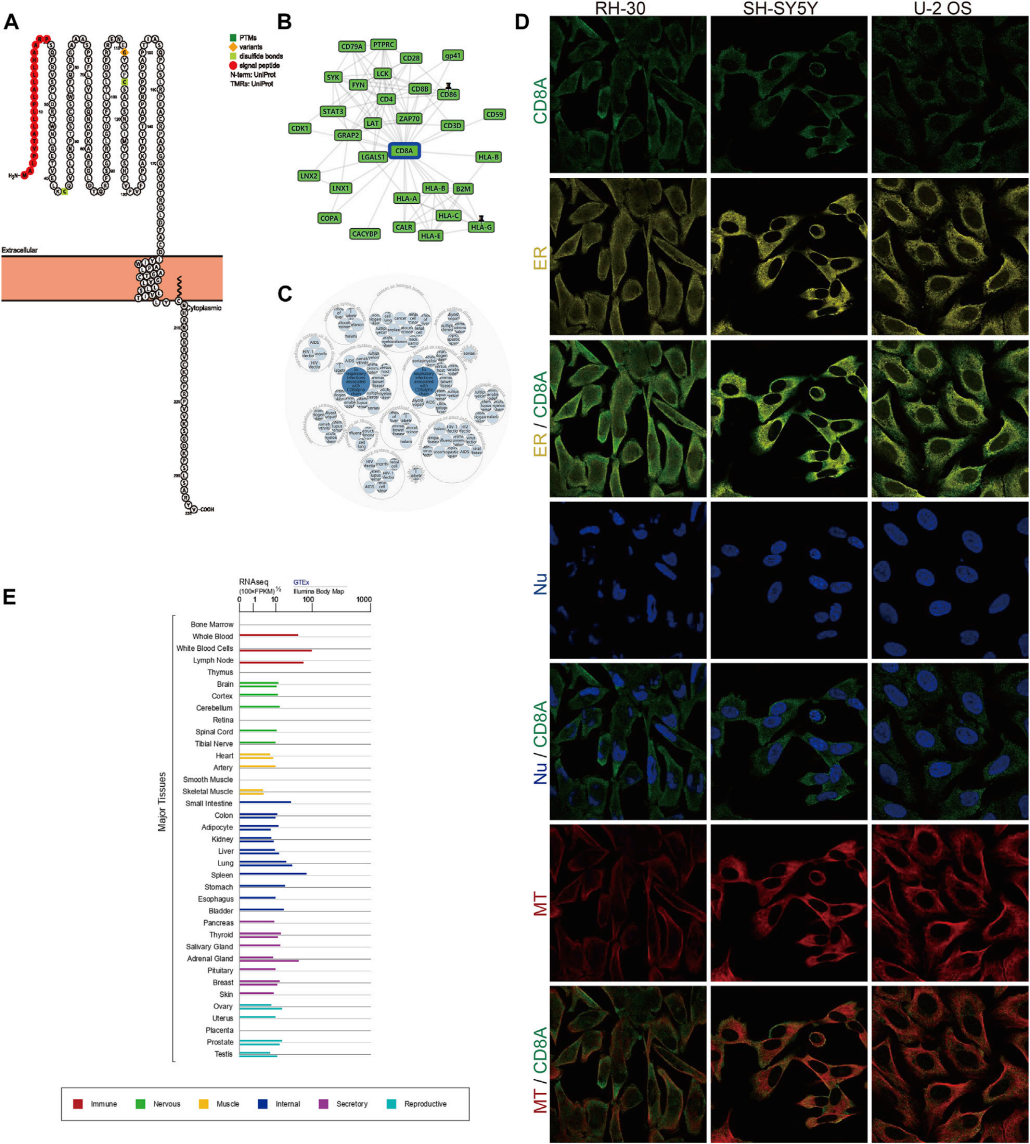
Protein topology, subcellular localization, PPI network analysis, and transcriptome expression of CD8A under normal physiological conditions **(A)** Protein topology of CD8A based on Protter database **(B)** PPI network construction of interacting genes with CD8A using GPS-Prot platform **(C)** Identification of diseases associated with CD8A via OPENTARGETS platform **(D)** Immunofluorescence analysis of cellular localization of CD8A in the alveolar rhabdomyosarcoma RH-30, human neuroblastoma SH-SY5Y, and human osteosarcoma U-2 OS cell lines through HPA database **(E)** The transcriptome profiles of CD8A in varying systems and organs of human body from GTEx database.

To deeply analyze the structural information of CD8A protein, we dissected annotated sequence features in the context of protein topology based on Protter database, manifesting the extracellular membrane structure of CD8A from Met1 to Pro21 as N-terminal signal peptide, as well as two disulfide bonds of Cys43, and Cys115. The natural missense variant, in particular, was localized to the extracellular membrane structure, exhibiting the amino acid change from glycine (G) to serine (S) at position 111, and plays a vital role in preventing CD8A expression, resulting in a complete deficit of CD8^+^ lymphocytes, as previously documented (Mancebo et al., 2008) (Figure 2A). In addition, PPI network analysis demonstrated interactions between CD8A and multiple immune-related genes (e.g., PTPRC, CD4, and HLA class genes) (Figure 2B). Notably, CD8A is correlated with diseases of various systems in the human body and genetic, familial, and congenital disorders (Figure 2C).

Next, we further performed indirect immunofluorescence analysis to clarify the cellular localization of CD8A. We evaluated distribution of CD8A was in the endoplasmic reticulum (ER) and microtubules (MT) in the alveolar rhabdomyosarcoma RH-30, human neuroblastoma SH-SY5Y, and human osteosarcoma U-2 OS cell lines, and we discovered that CD8A protein overlapped with ER and MT but was not expressed in the nucleus of 3 cell lines, indicating its subcellular location in the plasma membrane (Figure 2D). Intriguingly, the transcriptome profiles of CD8A differed in varying systems and organs and are most predominantly expressed in the immune system, reflecting its intimate involvement in immune-related activities (Figure 2E). In summary, we provided a detailed description of the transcriptomic and proteomic information of CD8A, presenting its multi-omic landscape under physiological status.

### Transcriptomic patterns and prognosis analysis of CD8A in pan-cancer datasets

**FIGURE 3:**
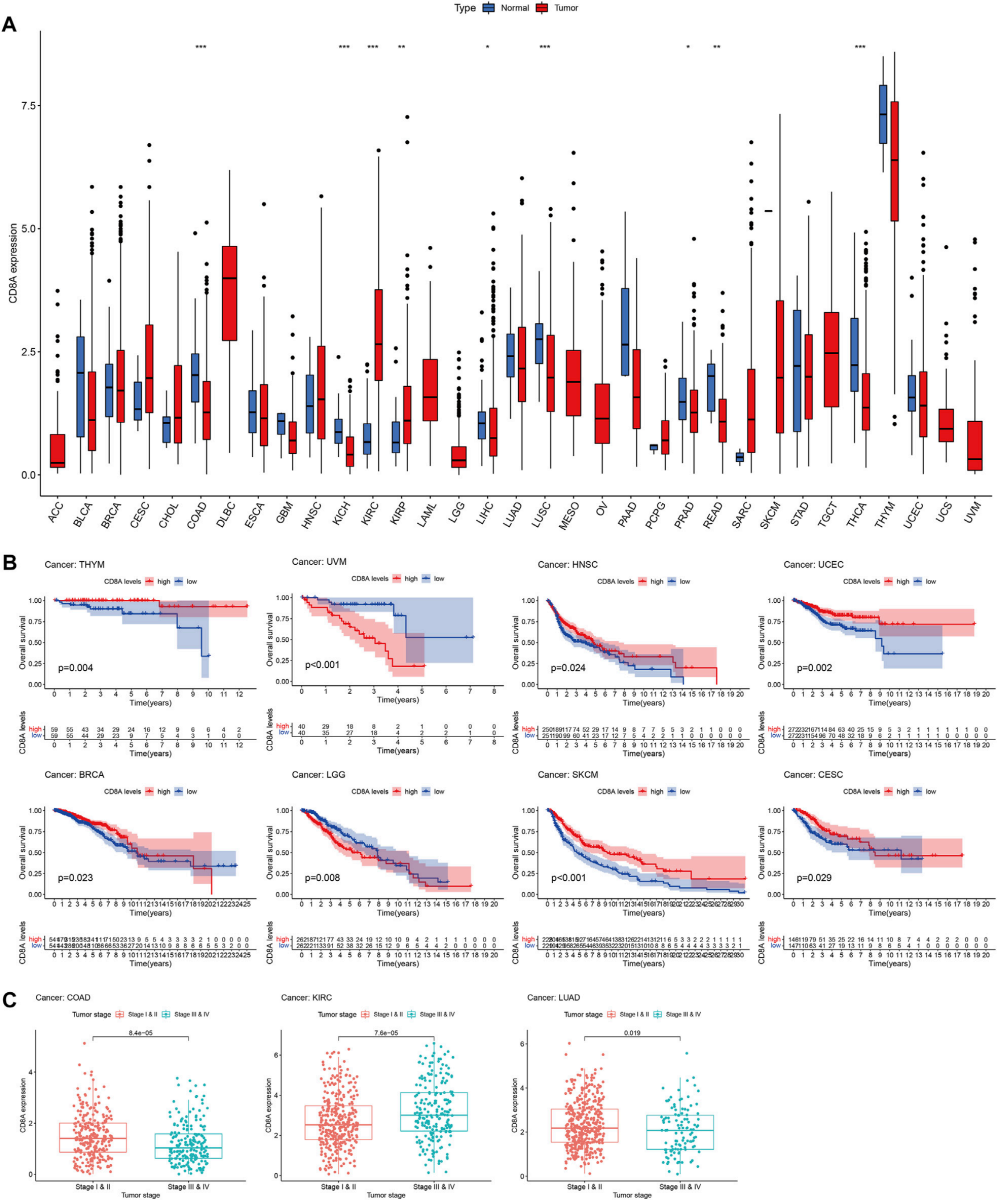
Transcriptomic patterns and prognosis analysis of CD8A in Pan-Cancer datasets **(A)** Analysis of CD8A expression patterns at transcriptomic level based on TCGA (^∗^ *p* < 0.05, ^∗∗^ *p* < 0.01, ^∗∗∗^ *p* < 0.001) **(B)** Correlation between CD8A expression and overall survival as determined through Kaplan–Meier curve analysis in pan-cancer datasets of TCGA **(C)** Correlation between CD8A and tumor stages in colon adenocarcinoma (COAD), kidney renal clear cell carcinoma (KIRC), and lung adenocarcinoma (LUAD).

We used TCGA database to perform a differential analysis of CD8A expression between cancer and normal tissues to score the transcriptomic patterns of CD8A. The findings revealed that CD8A expression was significantly diminished in varying cancer tissues, including colon adenocarcinoma (COAD), kidney chromophobe cell carcinoma (KICH), liver hepatocellular carcinoma (LIHC), lung squamous cell carcinoma (LUSC), prostate adenocarcinoma (PRAD), rectum adenocarcinoma (READ), and thyroid carcinoma when compared to normal controls (THCA). Conversely, the CD8A expression was enhanced in kidney renal clear cell carcinoma (KIRC) and kidney renal papillary cell carcinoma (KIRP) (Figure 3A). Following that, we dissected the correlation between CD8A expression level and the overall survival of pan-cancer patients, indicating that low CD8A expression may have deleterious effects on the prognosis of specific cancer types (Figure 3B), including thymoma (THYM), head and neck squamous cell carcinoma (HNSC), uterine corpus endometrial carcinoma (UCEC), BRCA, skin cutaneous melanoma (SKCM), cervical squamous cell carcinoma and endocervical adenocarcinoma (CESC). In contrast, low CD8A expression played a positive role in uveal melanoma (UVM) and brain low-grade glioma (LGG).

Besides, results elucidated that CD8A expression levels were closely linked to the tumor stage, as evident by higher expression levels in the early stage than in advanced stages of COAD and lung adenocarcinoma (LUAD). In contrast, higher expression levels in advanced KIRC were presented compared with the patients with stages I and II. The findings above shed light on the distinct CD8A expression patterns and their enormous potential as a robust biomarker for predicting prognosis and cancer stage (Figure 3C).

### CD8A is implicated in the formation of an immunosuppressive environment through T cell exclusion in TME of various cancer types, with the good predictive ability for immunotherapy efficacy

**FIGURE 4:**
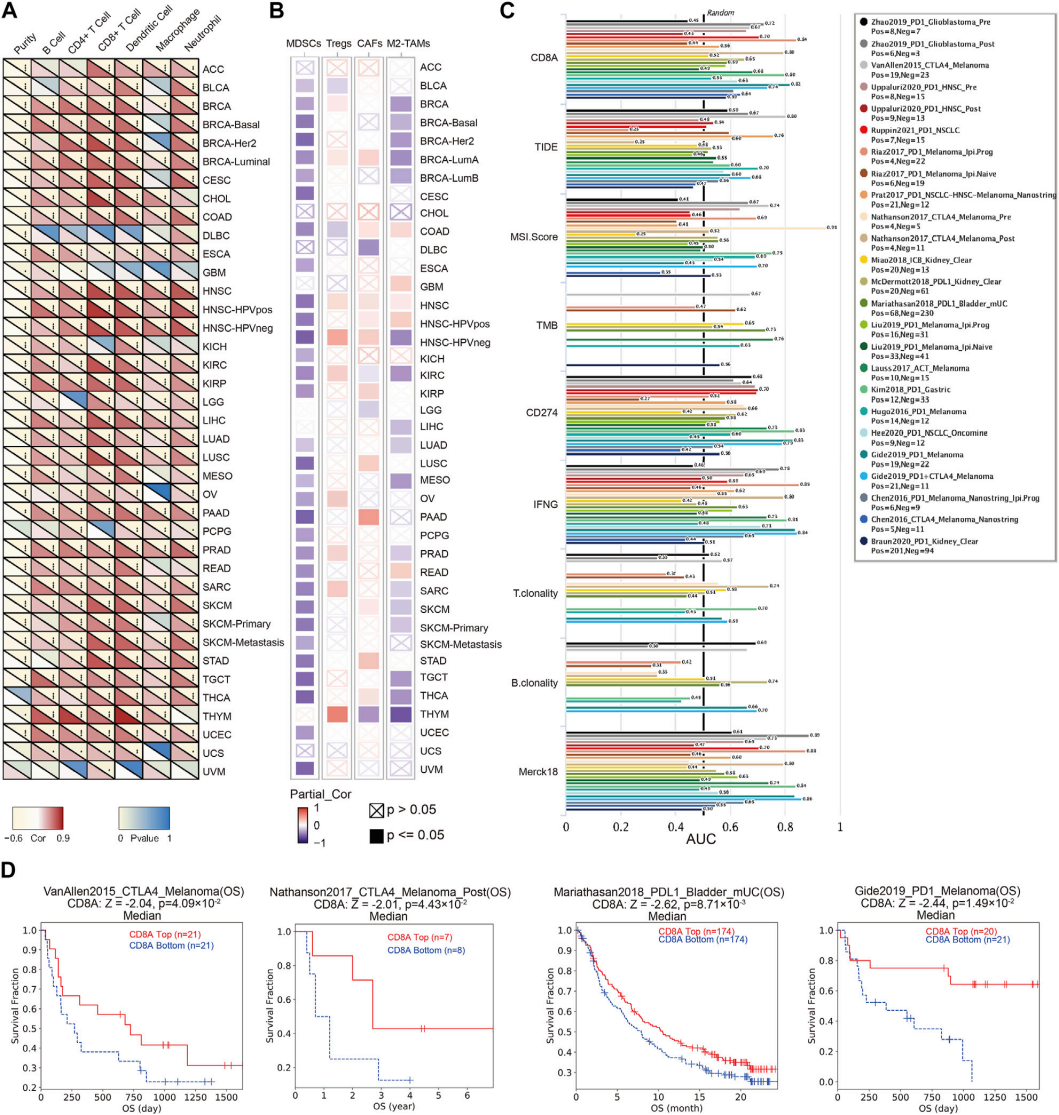
The role of CD8A in TME, and its potential in predicting the treatment responses of immunotherapy **(A)** Heatmap showing correlations between CD8A expression with six major immune cell types in the pan-cancer datasets based on TIMER2 server **(B)** Heatmap showing correlations between CD8A expression with four immunosuppressive cell in the pan-cancer datasets based on TIMER2 server**(C)** Bar plot presenting the predictive ability of CD8A for immunotherapy efficacy compared with other canonical biomarker signatures, based on treatment response, and prognosis of varying cancer cohorts treated with immune checkpoint blockade (ICB) in TIDE database **(D)** Correlation between CD8A expression and survival outcome of cancer cohorts treated with ICB, as well as its associations with cytotoxic T lymphocytes (CTL) levels.

TME is an ecological niche constituting various components, dynamically varying, which is associated with tumor progressions, and treatment response (Joyce and Fearon, 2015; Chen et al., 2021). Our results noted that CD8A were correlated with six major immune cell types in TME of multiple cancer types (Figure 4A), including BRCA, COAD, esophageal carcinoma (ESCA), HNSC, KIRC, KIRP, LIHC, LUAD, LUSC, pancreatic adenocarcinoma (PAAD), PRAD, sarcoma (SARC), SKCM, testicular germ cell tumor (TGCT), UCEC, and some of their specific cancer subtypes. Surprisingly, based on TIMER database, we discovered a consistently positive correlation between CD8A expression and all six major immune cells of aforementioned cancer types (R > 0, *p* < 0.05), indicating its critical role in reflecting the dynamical alterations of immune microenvironment remodeling.

Unambiguous evidence suggests that immunosuppressive cells account for T cell exclusion, thus inducing the establishment of the immunosuppressive microenvironment as a contributor to restriction for CTLs accumulation in the vicinity of tumor cells, which is considered a contributor to restriction as a reason for immunotherapy ineffectiveness (Garcia et al., 2014; Joyce and Fearon, 2015). Thus, we delineated the correlation analysis between CD8A and immunosuppressive cells, containing myeloid-derived suppressor cells (MDSCs), regulatory T cells (Tregs), cancer-associated fibroblasts (CAFs), and M2 subtypes of tumor-associated macrophages (M2-TAMs). Results indicated that CD8A were invariably negatively correlated with MDSCs in most cancer and subtypes, including bladder urothelial carcinoma (BLCA), BRCA, COAD, ESCA, HNSC, KIRC, KIRP, KICH, LUAD, LUSC, mesothelioma (MESO), ovarian serous cystadenocarcinoma (OV), PAAD, metastatic pheochromocytoma and paraganglioma (PCPG), PRAD, READ, SARC, SKCM, stomach adenocarcinoma (STAD), TGCT, THCA, UCEC, and UVM. Similarly, CD8A expression levels are also correlated negatively with M2-TAM in multiple cancer types, including BRCA, KIRC, LIHC, LUAD, MESO, PRAD, SARC, SKCM, TGCT, THCA, and THYM. In contrast, CD8A is positively correlated with Treg and CAFs in BRCA-LumA, HNSC, and HNSC-HPVneg.

Next, we compared CD8A’s predictive ability for immunotherapy efficacy to other canonical biomarker signatures in TIDE database, using treatment response and survival outcomes from various cancer cohorts treated with ICB. The results confirmed that CD8A had the highest predictive performance, with 21 of the 25 ICB-treated cohorts presenting an area under curve (AUC) greater than 0.5 (Figure 4C), comparable to CD274. Specifically, two melanoma cohorts presented the strongest predictive likeliness of responses for immunotherapy (Riaz2017_PD1 and Gide2019_PD1), with AUC values of 0.8409 and 0.8182, respectively, followed by glioma (AUC = 0.7222), non-small cell lung cancer [NSCLC (AUC = 0.7048)], and gastric cancer cohorts (AUC = 0.798), also presenting moderate predictive performances. Besides, as presented in Figure 4D, high CD8A expression was correlated to longer survival time of patients in CTLA4-treated melanoma cohort (VanAllen2015_CTLA4), PD1-treated melanoma cohort (Gide2019_PD1), PDL1-treated metastatic bladder cancer cohort (Mariathasan2018_PDL1), and PD1-treated NSCLC-HNSC-Melanoma cohort (Prat2017_PD1). Furthermore, we found invariably positive correlations between CD8A expression and CTL levels of patients in these cohorts, such observations corroborated the established role of CD8A in promoting CTL-mediated tumor killing (Oja et al., 2018; Krishna et al., 2021). Above results manifested that CD8A has the potential to be an indicator for TME remodeling, and its underlying impact on the formation of immunosuppressive microenvironments, as well as its good predictive ability for immunotherapy efficacy.

### Epigenetic modifications of CD8A were related to the CTL levels and T cell dysfunctional states, whereby affected survival outcomes of certain cancer types

**FIGURE 5:**
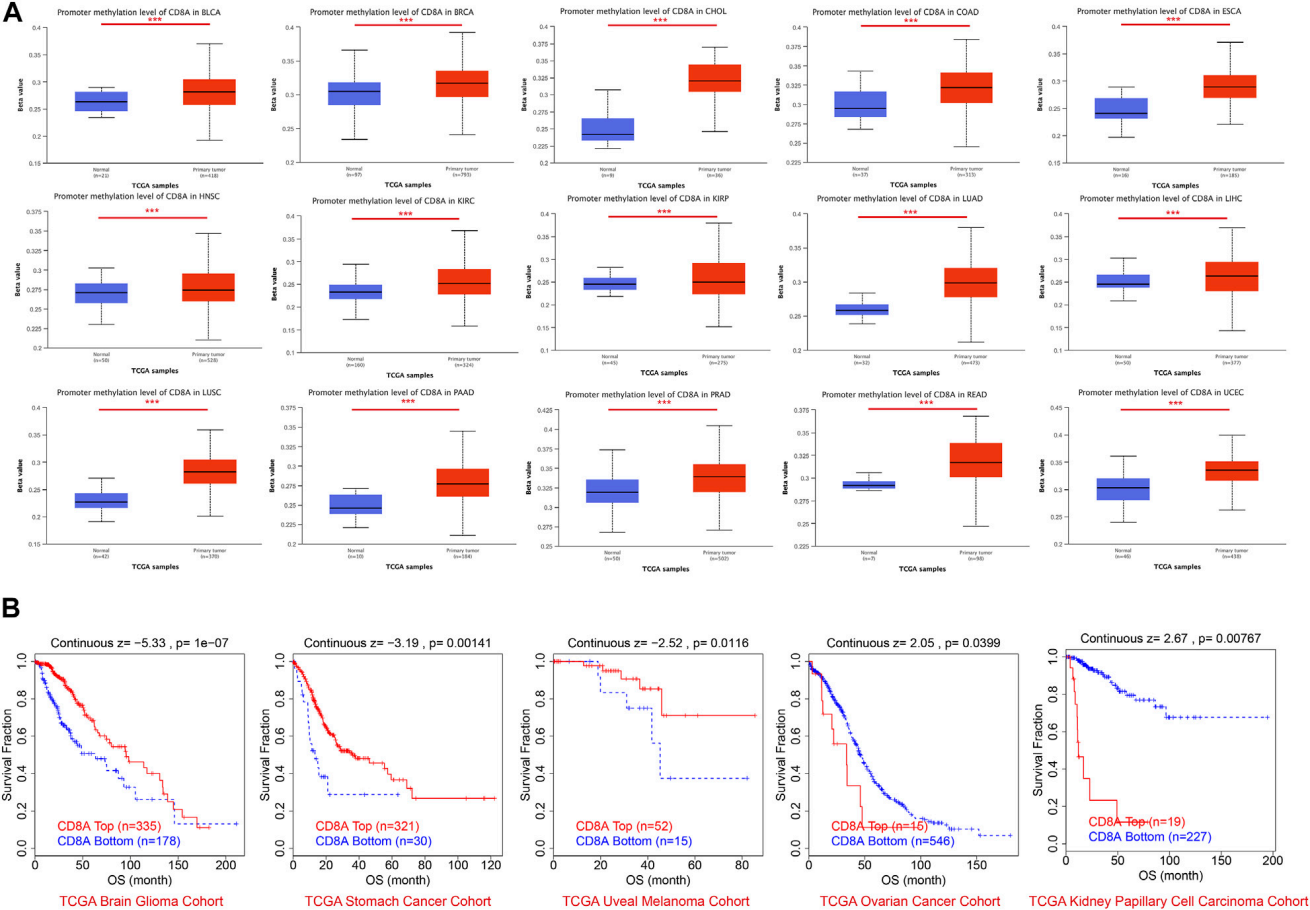
Epigenetic modification profiles of CD8A and its relationships with prognosis in pan-cancer datasets **(A)** Differential analysis of CD8A methylation levels based on UALCAN database **(B)** Correlation between CD8A methylation and prognosis as determined through Kaplan-Meier analysis based on TIDE database.

We also aimed to explore the relationship between the epigenetic modification status of CD8A and cancer patient prognosis, attempting to uncover deeper mechanisms of CD8A influencing tumorigenesis and treatment responses. The results disclosed that CD8A presented higher methylation levels in tumor tissues of multiple cancer types, including BLCA, BRCA, cholangiocarcinoma (CHOL), COAD, ESCA, HNSC, KIRC, KIRP, LUAD, LIHC, LUSC, PAAD, PRAD, READ, and UCEC, compared to its corresponding normal control (Figure 5A). Notwithstanding, according to the threshold, CD8A is invariably hypomethylated in BLCA, ESCA, HNSC, KIRC, KIRP, LUAD, LIHC, PAAD, and LUSC tissues. Concurrently, the remaining cancer types did not reach hypermethylation levels, indicating that epigenetic modifications in these cancer cohorts positively regulated their expression levels.

Survival analysis revealed that low methylation levels of CD8A were related to worse prognosis in glioma, gastric cancer, and uveal melanoma cohorts but were associated with longer survival outcomes in ovarian cancer and KIRP cohorts (Figure 5B). Notably, the methylation levels of CD8A manifested a significant negative correlation with level of CTLs in patients with glioma and uveal melanoma and a significant positive correlation with ovarian cancer cohort (Table 1). More importantly, Cox-PH model regression manifested the previously unappreciated association between CD8A and CTL level epigenetic modifications. In particular, we found an antagonistic interaction between CD8A methylation levels and CTL levels in uveal melanoma and KIRP cohorts (Table 1), corroborating its role in decreasing the beneficial association between CTL and survival outcome. In conclusion, above findings clarified the effect of CD8A epigenetic modifications on CTL levels and T cell dysfunctional states, which may contribute to the prognosis of patients with different tumor types, providing compelling evidence for disparities in opposite prognosis in various cancer types.

**TABLE 1:** Associations of CD8A epigenetic modifications with cytotoxic T lymphocytes (CTLs) levels, and T cell dysfunctional states, with Cox proportional hazard (Cox-PH) model regression presenting the role of CD8A methylations in influencing the interactions between CTL and survival outcome. ^∗^ *p* < 0.05, ^∗∗^ *p* < 0.01, ^∗∗∗^ *p* < 0.001.

Cohort	Cancer	Subtype	CTL cor	T dysfunction	Risk	Risk. Adj	Count
TCGA	Brain	Glioma	−0.25/***	0.677/ns	−5.326/***	−4.582	513
TCGA	Stomach	—	0.066/ns	0.903/ns	−3.192/**	−3.167	351
TCGA	Kidney	Papillary	−0.063/ns	2.216/*	2.666/**	2.731	246
TCGA	Uveal	—	−0.382/**	3.003/**	−2.524/*	−1.759	67
TCGA	Ovarian	—	0.091/*	0.162/ns	2.054/*	2.31	561

### Genomic alteration profiles, co-occurrence patterns of CD8A mutations, and its role in tumorigenesis

**FIGURE 6:**
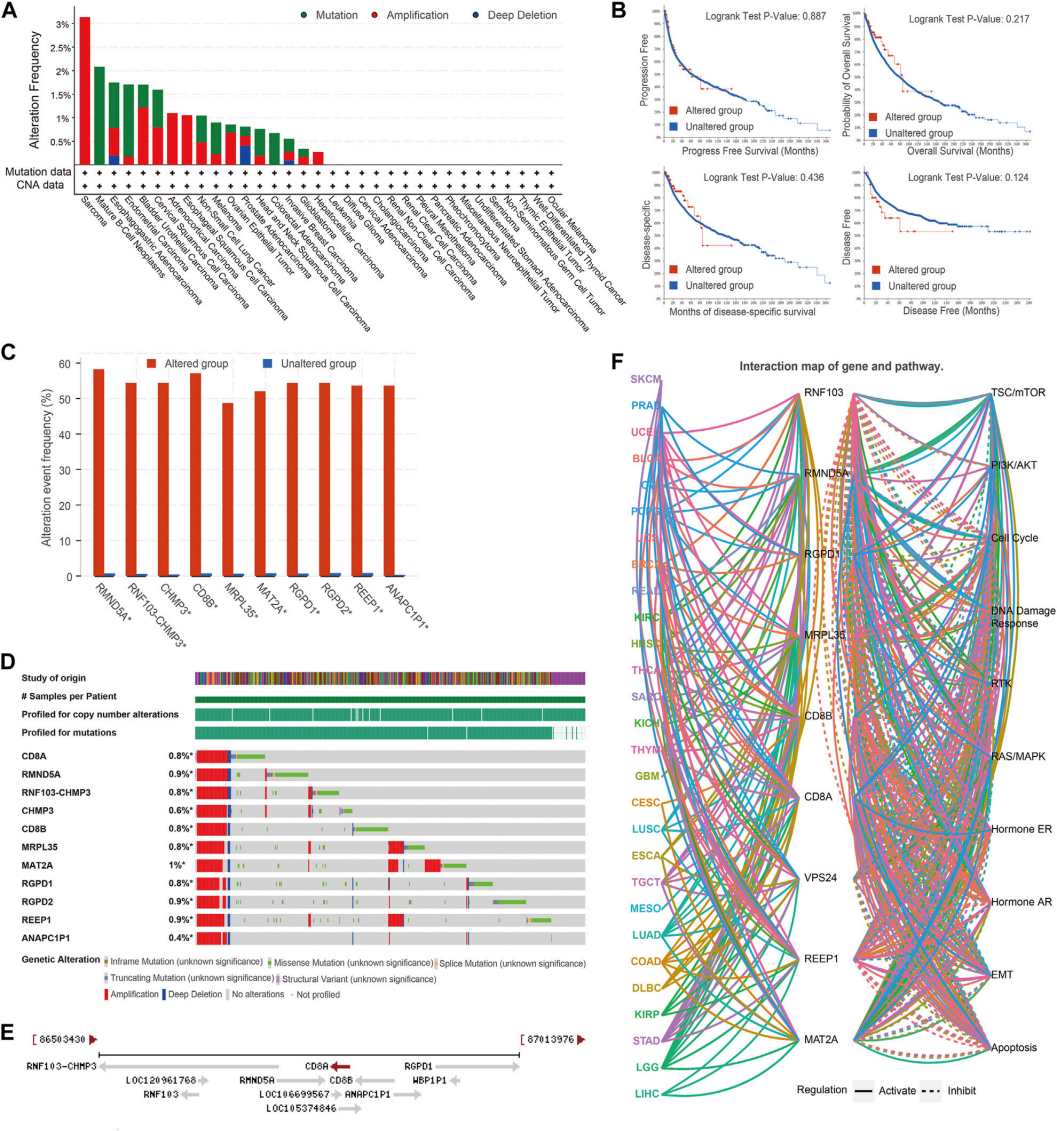
Genomic alteration profiles, co-occurrence patterns of CD8A mutations and its role in tumorigenesis **(A)** Genomic alteration profiles of CD8A in varying cancer types **(B)** Kaplan-Meier curves analyses of altered and unaltered CD8A groups of pan-cancer datasets in TCGA database **(C)** Co-occurrence frequency with CD8A alterations **(D)** Co-occurrence pattern of CD8A alterations **(E)** Chromosomal location of co-occurrence genes with CD8A **(F)** The role of co-occurrence genes in regulating cancer-related signaling pathways across various cancer types.

Next, we investigated genomic alteration profiles of CD8A in cancer, and results revealed that mutation and amplification were the most common alteration patterns (Figure 6A). Nonetheless, the survival indicators of patients with CD8A mutations did not differ significantly from those of non-mutated patients, namely in the altered and unaltered groups (Figure 6B). Thereafter, we explored the co-occurrence patterns of CD8A mutation and screened the top ten significant genes with log ration greater than 6, thereby identifying RMND5A, RNF103-CHMP3, CHMP3, CD8B, MRPL35, MAT2A, RGPD1, RGPD2, REEP1, and ANAPC1P1 genes, which co-occurred mutations with CD8A, sharing high mutation frequencies (Figure 6C), and genomic alterations (Figure 6D) in an altered group of pan-cancer datasets. Intriguingly, the chromosomal location of these genes is very similar to that of CD8A (Figure 6E). The highly identical genomic alteration profiles and chromosomal location suggest their interactively synergetic roles in physiological and pathological processes. Thus, we reasoned that there was an interaction between these genes and tumorigenesis. The genes mentioned above regulate cancer-related signaling pathways in various cancer types, including the phosphatidylinositol 3-kinase (PI3K)/protein kinase B (AKT), epithelial-mesenchymal transition (EMT), DNA damage response pathways, estrogen and androgen receptor-related pathways (Figure 6F).

### CD8A as a therapeutic biomarker indicating multiple antitumor agents’ responsiveness on various cancer cell lines and cancer cohorts

**FIGURE 7:**
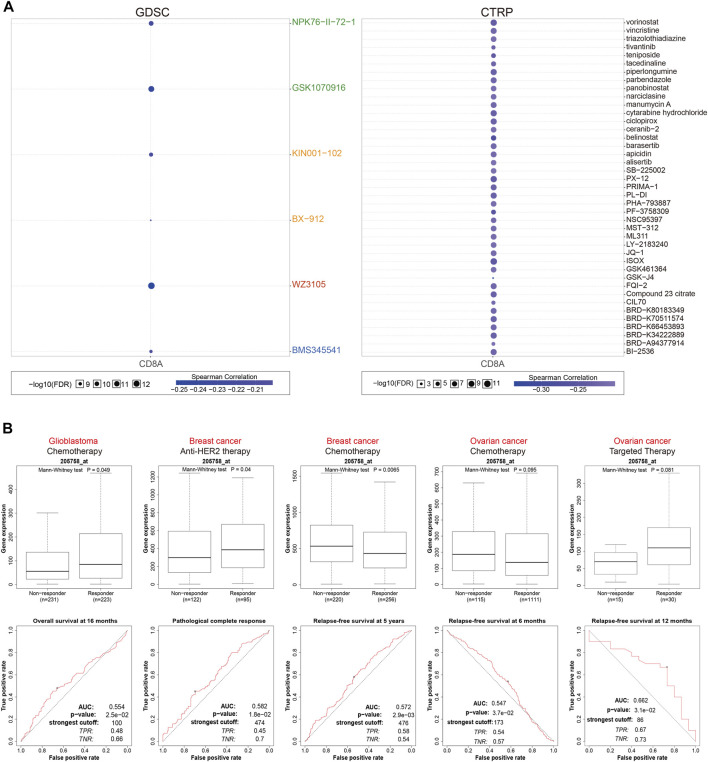
CD8A as a therapeutic biomarker indicating multiple anti-tumor agents’ responsiveness on various cancer cell lines, and cancer cohorts **(A)** Associations between CD8A expression, and 50% inhibitory concentration (IC_50_) values of varying antitumor agents or compounds in tumor cell lines based on GDSC and CTRP databases **(B)** Associations between the expression levels of CD8A, and therapeutic responses to various antitumor drugs in clinical cancer cohorts.

Based on the GDSC database, we then examined associations between CD8A and sensitivity and responsiveness of tumor cell lines to various antitumor agents or compounds (Figure 7A). Interestingly, results indicated that CD8A expression levels were inversely correlated with the IC_50_ of different antitumor agents to tumor cell lines, including several previously reported drugs; aurora kinase (AURK) inhibitor: GSK-1070916 (Hardwicke et al., 2009), 3-phosphoinositide-dependent protein kinase-1 (PDK-1) inhibitor: BX-912 (Bai et al., 2021), and nuclear factor kappa B inhibitor: BMS345541 (Li et al., 2019). Notably, the results from CTRP database demonstrated significant negative correlations between CD8A. IC_50_ values of 41 types of classical chemotherapeutics or targeted drugs approved by FDA, containing histone deacetylase (HDAC) inhibitor: vorinostat (Siegel et al., 2009), microtubule assembly inhibitor: vincristine (Hawkins et al., 2018), and topoisomerase II inhibitor: teniposide (Liu L. et al., 2018), DNA damage inducer: cytarabine (Qi et al., 2020), which manifest that high CD8A expression was related to the decreased sensitives and efficacy of such agents (Figure 7B).

Following that, we investigated the utility of CD8A in evaluating therapeutic responses to various antitumor drugs in clinical cancer cohorts (Figure 7C). In GBM treated with chemotherapy, CD8A expression level was higher in responders, and the time-dependent ROC curves analysis revealed that CD8A had a good predictive ability of overall survival at 16 months, with an AUC of 0.554. Likewise, CD8A was associated with benefits of anti-HER2 therapeutic pathological complete response and chemotherapeutic relapse-free survival (RFS) at 5 years in the BRCA cohorts, with AUC of 0.582 and 0.572, respectively, while the non-responders presented augmented CD8A expression levels relative to responders. AUC of ROC curve for predicting 6- and 12-months RFS in ovarian cancer cohorts treated with chemotherapy and targeted therapy were 0.547 and 0.662, respectively. CD8A expression levels did not differ significantly between responders and non-responders. Taken together, above results elucidated that CD8A could efficaciously function as a therapeutic response biomarker in various cancer cell lines and cohorts treated with multiple anticancer agents.

### Single-cell transcriptomics revealed the role of CD8A in TME, as well as its relevance across functional states in distinct cancers

**FIGURE 8:**
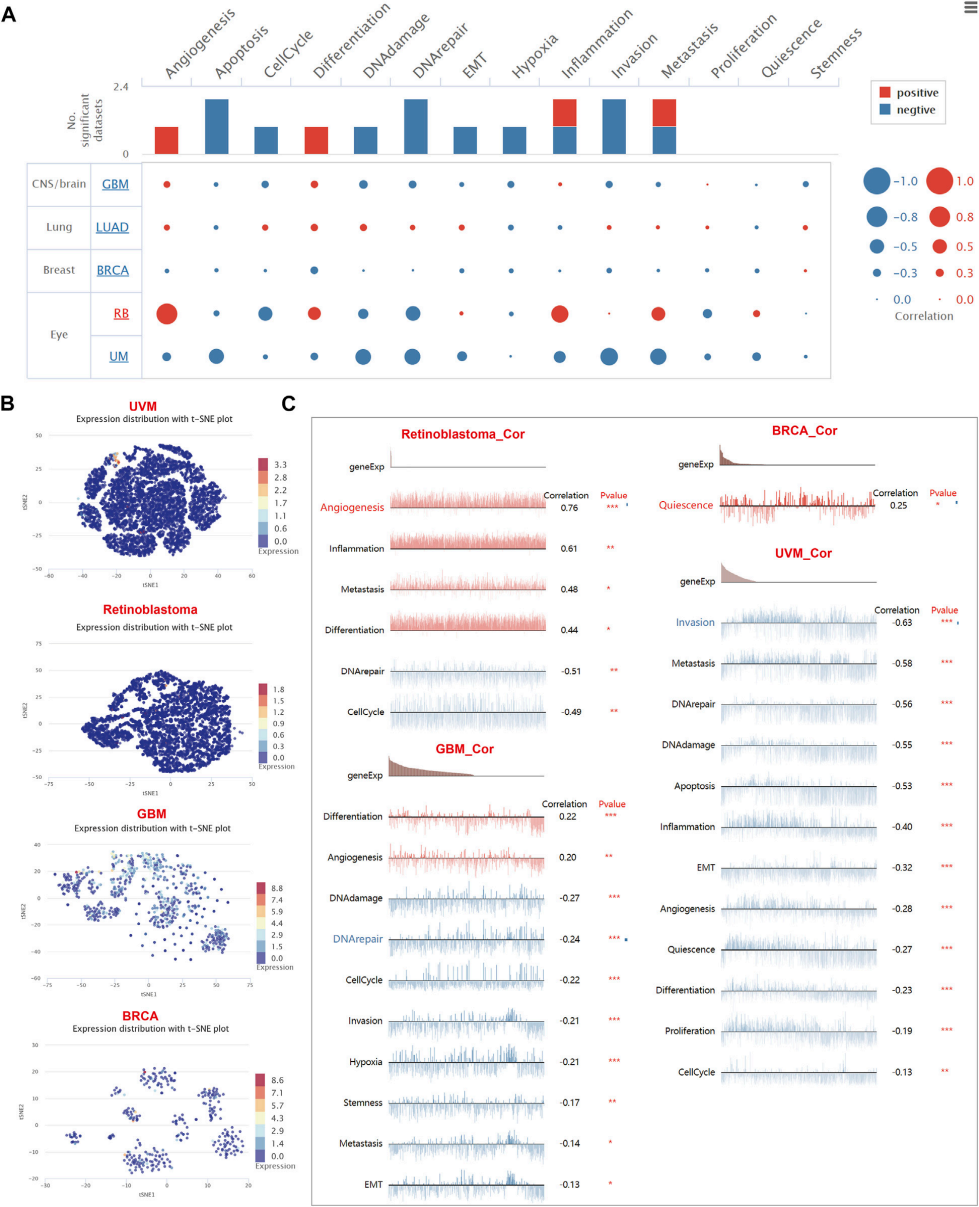
The role of CD8A and its functional relevance across varying cancer types at single-cell resolution **(A)** Correlations between CD8A and functional relevance of varying cancer types, with size of the bubble indicating strength of correlation, and bar plots showing datasets number in which CD8A significantly related to the corresponding state **(B)** Single-cell clustering visualization using tSNE disclosed the heterogeneity in the expression levels of CD8A in various cell clusters **(C)** Correlation between CD8A expression, and functional states in distinct cancers.

Then, we aimed to interrogate the role of CD8A in TME of diverse cancer types at single-cell resolution and its correlation with malignant phenotype and functional states. Thus, we focused on expression levels of CD8A in published single-cell data from five cancer types, including GBM (Patel et al., 2014), LUAD (Kim et al., 2015), BRCA (Chung et al., 2017), retinoblastoma (RB) (Liu et al., 2020), and UVM (Durante et al., 2020), with results illustrating that CD8A was significantly associated with multiple malignant phenotypes and functional states of four cancer types except for LUAD (Figure 8A).

The results of single-cell clustering visualization using t-distributed stochastic neighbor embedding (tSNE) of above-mentioned cancers revealed that the heterogeneity in the expression levels of CD8A in various cell clusters of GBM was found to be mild (Figure 8B), and the correlation analysis indicated that CD8A were positively correlated with malignant phenotypes of differentiation and angiogenesis. Concurrently, it showed a negative correlation with the other eight functional states (Figure 8C), including DNA damage, DNA repair, cell cycle, invasion, hypoxia, stemness, metastasis, and EMT. Interestingly, we found that CD8A presented consistently low expression levels in TME of the other four cancer types (Figure 8B) and was only expressed in a small portion of cells. Furthermore, in retinoblastoma and UVM, CD8A manifested significant negative correlations with two and twelve functional states, respectively, while only showing a positive correlation with quiescence in BRCA (Figure 8C).

## Discussion

We tested whether CD8A could be a reliable prognostic biomarker predicting survival outcome of cancer patients in this study, and results based on pan-cancer datasets in TCGA database revealed its aberrant expression profiles in cancer tissues were tightly associated with patient’s clinical characteristics as well as disease phenotypes. Furthermore, our conclusions shed light on the potential roles of CD8A in immunotherapy. Although multiple biomarkers have been proposed to predict the therapeutic response of ICBs, different degrees of limitations exist in clinical applications. Immunohistochemistry (IHC)-based PD-L1 detection was the first candidate indicator proposed in this field (Patel and Kurzrock, 2015), as evidenced by studies of Taube (Taube et al., 2014) and Borghaei (Borghaei et al., 2015), reporting that PD-L1 expression levels presented good predictive ability in reflecting treatment responses to malignancies in patients treated with nivolumab, similar results also obtained in NSCLS cohorts treated with pembrolizumab (Garon et al., 2015). Despite this, some studies have found contradictory results, indicating that PD-L1 expression was not associated with nivolumab efficacy in advanced renal cell carcinoma patients (Motzer et al., 2015). In fact, opposite results were even reported in the Checkmate 037 (Weber et al., 2015) and Checkmate 066 (Robert et al., 2015) trials, demonstrating that patients negative for PD-L1 expressions still have better treatment responses. Lessons learned over the past regarding implementing IHC-based PD-L1 detections elucidated the existence of significant discordance rates when varied antibody used, disparate cells were stained, and the different cut-off values were chosen in the clinical setting (Fusi et al., 2015).

In this study, we explored CD8A as a biomarker for predicting response of ICBs. CD8A exhibited good predictive performance, with 21 of 25 ICB-treated cohorts having an AUC greater than 0.5 (Figure 4C), among which two melanoma cohorts exhibited the strongest predictive likeliness for PD1 blockade. Cancer patients with higher expression levels of CD8A could derive better survival outcomes when treated with anti-PD1 or anti-CTLA4, in accordance with the seminal studies of Tumeh and others (Tumeh et al., 2014), indicating that metastatic melanoma patients responding to pembrolizumab were characterized by proliferation of intra-tumoral CD8^+^ cells that directly correlated with a radiographic reduction in tumor size, with further multivariate analysis manifesting that CD8^+^ density in the invasive margin adjacent to the tumor as the best entire predictive parameter. Furthermore, genetic signatures have been proposed to predict the therapeutic benefit of ICBs using RNA-seq data retrospectively, among which CD8A is a critical cardinal ingredient. Sangro *et al.* (Sangro et al., 2020) conducted a retrospective analysis using CD8A-related signatures originating from prior literature, including the 4-gene inflammatory signature (Ayers et al., 2017; Siemers et al., 2017), and the Gajewski 13-gene inflammatory signature (Spranger et al., 2015), with results indicating that the signatures were closely associated with improved objective response rate (ORR) and overall survival to nivolumab therapy of hepatocellular carcinoma patients in the CheckMate 040 study. Notably, our study demonstrated the predictive potential of CD8A in antitumor compounds or agents, with the results based on GDSC and CTRP databases uniformly presenting close correlations between CD8A and IC_50_ of such agents to cancer cell lines. More importantly, we discovered that CD8A is a response predictor of chemotherapy and anti-HER2 therapy, which is consistent with report of Denkert (Denkert et al., 2015) and Bianchini (Bianchini et al., 2015). As a result, we may be able to elucidate CD8A’s significant potential as a response predictor to various antitumor therapies, which may aid in understanding the mechanisms underlying drug resistance and survival outcome differences in certain cancer patients.

TME is a disparate ecological niche, not only composed of heterogeneous neoplastic cells but stromal and immune cells (Chen et al., 2021). Dynamic interactions between neoplastic cells and other components could exert either tumor-suppressive or tumor-promoting effects (Turley et al., 2015; Salmon et al., 2019). “Allies,” represented by CD8^+^ and CD4^+^ T cells, together orchestrate an efficient antitumor immunity. The former initiates cytotoxic reactions that cause the death of neoplastic cells (Bejarano et al., 2021), and the latter coordinates adaptive immune responses by secreting a wide range of effector cytokines (van den Broek et al., 2018; Salemme et al., 2021). Correspondingly, “hostiles,” namely specific immune sub-populations (viz., Treg, M2-TAM, and MDSC) and stromal components (viz., CAF), could blunt the host antitumor immune response via the productions of cytokine and soluble factors (Salemme et al., 2021), among which MDSC takes the center stages in this circumstances (Tian et al., 2019), characterized by secreting high levels of suppressive molecules [e.g., reactive oxygen species (ROS), inducible nitric oxide synthase (iNOS)] (Condamine et al., 2015; He et al., 2018), recruiting Tregs (Salemme et al., 2021), and facilitating the differentiation of M2-TAMs (Weber et al., 2018). Notably, understanding the immunosuppressive TME contributes to tailor therapeutic regimens that may sensitize cancers to anticancer therapy, thus providing a framework for investigating cell-cell crosstalk and drug efficacy in tumor ecosystems (Koikawa et al., 2021).

Herein, we have elucidated the essential role of CD8A in TME, thus revealing the associations between CD8A and the aforementioned cell subpopulations. Specifically, we found a convergently positive correlation between CD8A and all six major types of immune cells of the pan-cancer TME. These results are unsurprising, as CD8A, *per se*, has been considered an indicator of immune cell infiltration (Chen et al., 2017). Despite being expressed in other immune cell types (e.g., natural killer T cells or dendritic cells) (Chen et al., 2017; Kim et al., 2019), CTLs have the most dominant expression level of CD8A, which could be directly indicative of pre-existing antitumor immunity in TME (Lei et al., 2021). Nonetheless, the interactions of CD8A with immunosuppressive cells have not been well characterized previously. Thus, we investigated the role of CD8A in the context of immunosuppressive TME, with correlation analysis revealing that it has close associations with immunosuppressive cells. We speculated that this tremendously interesting connection results from the negative crosstalk of CTL with such specific cell types, as corroborated by Xiang (Xiang et al., 2018) and Genard (Genard et al., 2018), indicating that IFN-γ and TNF-α produced by CTL could be a potent anti-M2 polarizing cytokine that closely related to M1 phenotype. Similarly, MDSC has been shown to suppress CD8^+^ T cell activation and proliferation (Tavazoie et al., 2018), explaining some of the negative crosstalk between MDSC and CTLs. Surprisingly, correlations between CD8A and Treg and CAFs varied by cancer type, and the underlying mechanism for this ambiguous phenomenon may be due to the heterogeneity of TME of pan-cancers.

Although previously published transcriptome studies on cancer have drawn magnificent significance in guiding cancer management, traditional molecular biology techniques, such as bulk RNA sequencing data, only provide interpretation of the averaged gene expression at the populational level, masking the slight yet vital information observable in cellular levels (Zou et al., 2021). Single-cell RNA sequencing (scRNA-seq) has broadened our horizons in exploring physiological and pathological transcriptomic changes at higher resolutions (Ma S. et al., 2020; Liao et al., 2020). In this work, we investigated functional identity information of CD8A at single-cell resolution, with tSNE plots originating from multiple cancer datasets manifesting that CD8A consistently exhibited low expression levels in TME. In contrast, comparatively high levels of CD8A expression were shown in the GBM dataset. Interestingly, we noticed a consistent negative correlation between CD8A, and various functional states, including invasion, metastasis, EMT, angiogenesis, and proliferation, of UVM dataset published by Durante *et al.*, (Durante et al., 2020), whose study indicated that elevated expression levels of CD8A accompanied CD8^+^ T cells in UVM TME, and specific immune checkpoint molecule LAG3. Nonetheless, other immune checkpoint molecules, PD1, CTLA4, TIM3, and TNFRSF9, only presented low expression levels, which may be the reason for the unsatisfactory therapeutic efficacy of anti-PD1, and CTLA4, thus revealing the great potential of anti-LAG3 in UVM. The results above denoted that CD8A manifested a negative correlation with multiple cancer-related functional states, indicating that CD8A exerts antitumor effects and maintains cancer immunity in UVM. Confusingly, other cancer types showed mixed results, with complex associations with varying functional states. Similarly, CD8A-related scRNA-derived signatures have demonstrated remarkable performance in predicting response effects in antitumor drugs, as demonstrated by Krishna and others (Krishna et al., 2021). They discovered strong enrichment of the CD8A + tissue-resident T cell cluster across all tumor regions, along with low TAM infiltration in TME of patient complete response to ICBs, thereby establishing a CD8A + tissue-resident signature that was associated with improved efficacy with patients treated with ICBs and targeted therapy. Despite certain essential discoveries revealed by our study, there are some limitations. First, it should be noted that we have carried out the analysis only from publicly available databases, and further *in vivo* or *in vitro* experiments remain imperative to validate the results obtained. Second, although the correlation between CD8A and various components of TME can surface from the data, it has not been indicated that the mechanism by which CD8A affects such cell populations.

## Conclusion

Overall, our study convincingly demonstrated that CD8A has enormous potential as a robust biomarker predicting cancer patient survival outcome and their clinical stage, with additional data indicating that CD8A was implicated in forming an immunosuppressive environment through T cell exclusion in TME. We dissected the epigenetic modifications, genomic alteration profiles, co-occurrence patterns of CD8A, and its role in tumorigenesis, proving its good predictive ability for multiple anticancer therapies. We aimed to uncover the underlying mechanisms and predictive accuracy of CD8A, thereby laying the groundwork for future cancer management and research.

## Data Availability

The original contributions presented in the study are included in the article/supplementary material, further inquiries can be directed to the corresponding authors.
